# Rotaxanes with integrated photoswitches: design principles, functional behavior, and emerging applications

**DOI:** 10.3762/bjoc.21.179

**Published:** 2025-10-31

**Authors:** Jullyane Emi Matsushima, Khushbu, Zuliah Abdulsalam, Udyogi Navodya Kulathilaka Conthagamage, Víctor García-López

**Affiliations:** 1 Department of Chemistry, Louisiana State University, Baton Rouge, LA 70803, USA

**Keywords:** macrocycle, photoisomerization, photoswitches, rotaxanes, shuttling

## Abstract

Integrating molecular photoswitches into rotaxanes offers unique opportunities for precise control over their structural, dynamic, and functional properties. By harnessing light as a non-invasive stimulus with high spatial and temporal resolution, these photoswitches allow for the modulation of the rotaxanes’ intra- and intermolecular interactions, optoelectronic properties, and shuttling dynamics. In this review, we discuss key examples of photoswitchable rotaxanes, organized according to the position of the photoswitch, either embedded in the axle or incorporated into the macrocycle. We examine the major classes of photoswitches used, including their switching mechanisms and the resulting influence on rotaxane operation. Due to their architectural versatility and precise light control, photoswitchable rotaxanes hold promise for a broad range of applications, including light-responsive molecular machines, smart materials, and biofunctional systems. However, emerging applications increasingly require rethinking and developing new structural designs that incorporate more efficient and advanced photoswitches to fully realize their potential.

## Introduction

Harnessing light energy to control intra- and intermolecular interactions is a powerful strategy for designing sophisticated molecular systems that operate across multiple length scales, from nanoscale dynamics to macroscopic functions [1–3]. Light is particularly attractive compared to other stimuli due to its non-invasive nature, high spatial and temporal precision in activating systems, and the ability to fine-tune responses by adjusting wavelength, intensity, and exposure time. Additionally, photoswitchable molecules can often operate reversibly without generating chemical waste or requiring the addition of chemical fuels [4–5].

**Figure 1:**
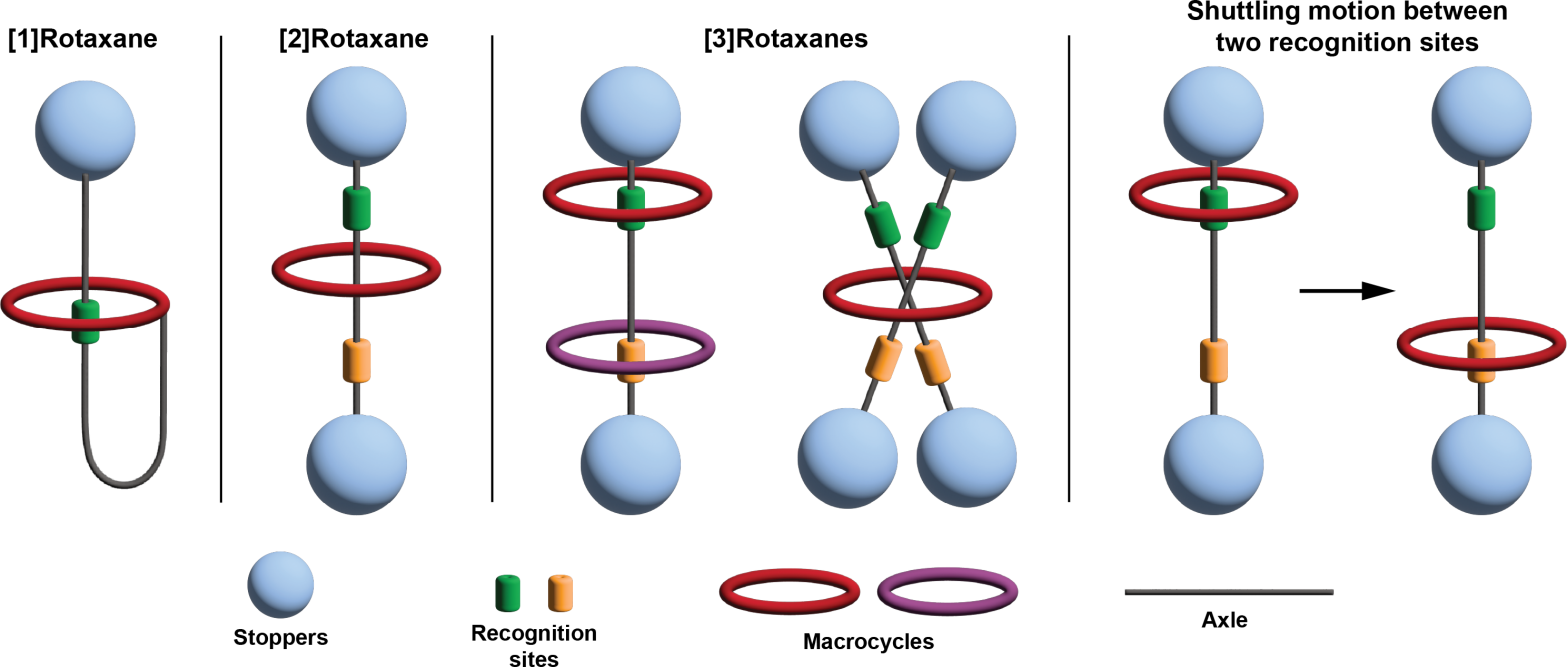
Schematic of common rotaxanes (left) and depiction of the macrocycle shuttling (right).

In the last three decades, photoswitches have been successfully integrated into a variety of supramolecular architectures, including mechanically interlocked molecules (MIMs) such as rotaxanes, enabling new dynamic functions [6–7]. Rotaxanes are molecules composed of a macrocycle threaded onto a linear axle, with bulky stoppers at both ends to prevent dethreading (Figure 1) [1,8]. Functionalization of the axle with recognition sites allows the macrocycle to shuttle between positions in response to external stimuli, including light, redox, temperature, pH, and reactions with chemicals such as carbodiimides [9–12].

Depending on their structure, rotaxanes can be classified by the number of components involved. In [1]rotaxanes, the macrocycle is covalently attached to one end of the axle while remaining mechanically interlocked with another part of the axle (Figure 1) [13–15]. In contrast, [2]rotaxanes consist of a macrocycle and an axle held together purely by mechanical bonding without covalent linkage. More complex architectures, such as [3]rotaxanes, may feature two macrocycles threaded onto a single axle or one macrocycle threaded onto two axles (Figure 1) [16–19].

Integrating photoswitches into rotaxane frameworks has resulted in a rapidly expanding class of systems capable of light-driven molecular motion, controlled conformational changes, and tunable functional outputs. Consequently, photoswitchable rotaxanes provide unique design opportunities for a broad range of applications, including responsive materials, sensing, cargo delivery, information storage, and biotechnology [7,16–17,20–23].

**Figure 2:**
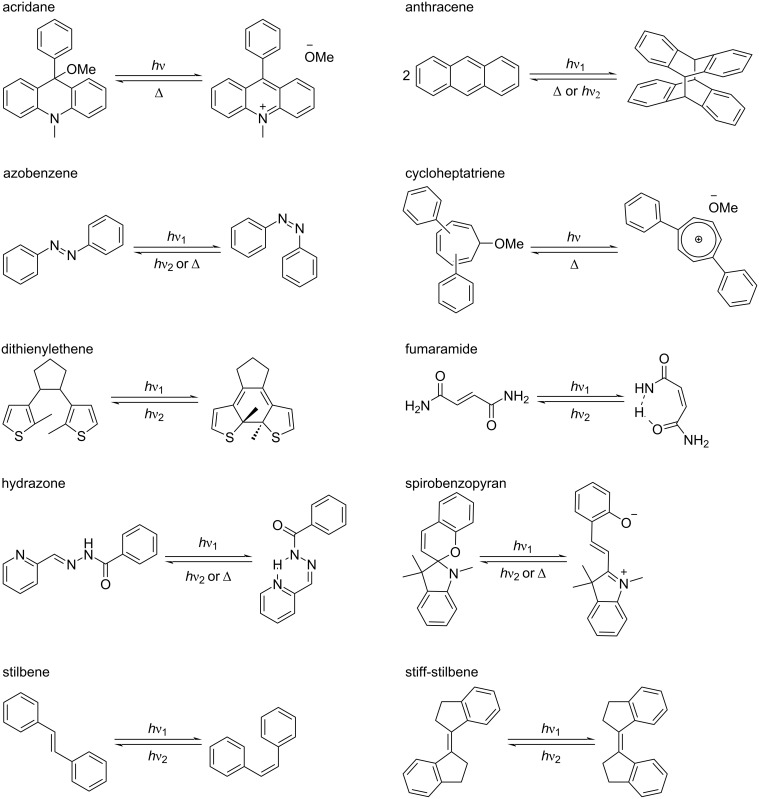
Structure of some common photoswitches integrated into rotaxanes.

In this review, we broadly discuss the design principles, functional behaviors, and emerging applications of rotaxanes that have integrated photoswitches. We classify key examples based on the location of the photoswitch within the rotaxane structure – either integrated into the axle or situated in the macrocycle. Within these categories, we highlight widely used photoswitches, including acridane, anthracene, azobenzene, cycloheptatriene, dithienylethene, fumaramide, hydrazone, spirobenzopyran, stilbene and stiff-stilbene (Figure 2). Systems whose photoinduced behavior primarily depends on photoredox or energy-transfer mechanisms fall outside the scope of this review.

## Review

### Rotaxanes featuring photoswitches on the axle

Rotaxanes that incorporate photoswitchable units into the axle are the most extensively developed and studied class of photoswitchable rotaxanes, in contrast to those in which the photoswitch is integrated into the macrocycle. Incorporating a photoswitch into the axle is used to modulate the non-covalent interactions between the macrocycle and the axle, thereby regulating the macrocycle’s position and, when appropriate, its shuttling rate. This section highlights representative examples featuring the most widely used photoswitches.

#### Acridane

Acridane photoswitching involves its photoheterolysis, generating a positively charged acridinium and an alkoxide, typically a methoxide anion (Figure 2). The neutral acridane is restored through the thermal nucleophilic attack of the alkoxide on the acridinium ion. Abraham and co-workers exploited this dramatic change in the electronic nature and geometric shape of acridane to modulate the translation of cyclobis(paraquat-4,4-bisphenylene) (CBPQT^4+^) along the axle of rotaxanes [24]. Specifically, they used acridane as a recognition unit and stopper in a [2]rotaxane. Thus, the electron-rich acridane interacts with the electron-poor CBPQT^4+^, and upon light irradiation, the formation of the cationic acridinium repels the CBPQT^4+^ macrocycle and promotes its translation away from the acridane toward the secondary site. However, if the rotaxane contains only one recognition site, the macrocycle remains at the unfavorable acridinium station, interacting with the 9-aryl group. Therefore, substitution at this position was employed to tune the macrocycle interaction with the axle.

**Figure 3:**
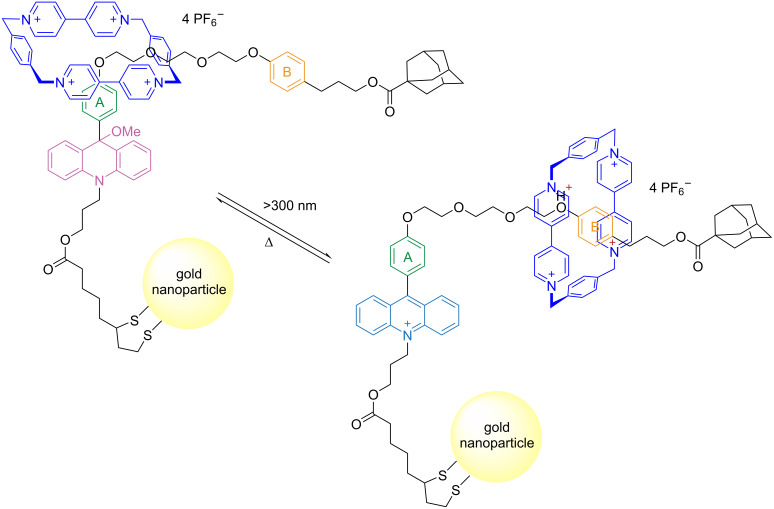
Rotaxane with an acridane photoswitch on the axle modulates the translation of a CBQT4^+^ macrocycle on gold nanoparticles. Figure 3 was adapted with permission of The Royal Society of Chemistry, from [21] (“Photoswitchable rotaxanes on gold nanoparticles” by Y. Duo et al., *Org. Biomol. Chem.*, vol. 9, issue 9, © 2011); permission conveyed through Copyright Clearance Center, Inc. This content is not subject to CC BY 4.0.

In a different approach, the authors demonstrated that the macrocycle can shuttle along an axle by incorporating two acridane photoswitches at both ends. However, upon light activation, the macrocycle stops shuttling and remains in the middle of the axle, thereby avoiding the terminal cationic sites [25]. The shuttling is restored by the thermal reaction that regenerates the neutral acridane [24–26]. The same group also demonstrated that similar rotaxanes can be incorporated onto gold nanoparticles and retain their photoreactivity and switching behavior, enabling the translation of the macrocycle along the axle upon irradiation with light (Figure 3) [21].

#### Anthracene

Anthracene undergoes [4π + 4π] dimerization upon irradiation with light, whereas cycloreversion is achieved thermally (Figure 2) [27]. Tron and co-workers synthesized a [2]rotaxane composed of a dibenzo-24-crown-8 (DB24C8) and an axle with anthracenes as stoppers on both ends [28]. Light irradiation at 365 nm triggered the dimerization of the anthracenes allowing the conversion of the [2]rotaxane into a [2]catenane. Cycloreversion and formation of the rotaxane was achieved thermally or upon irradiation with light at 280 nm.

#### Azobenzene

Azobenzene is one of the most widely utilized photoresponsive units in the design and synthesis of photoswitchable rotaxanes. These photoswitches undergo *trans*–*cis* isomerization and are classified as T-type, due to the thermal reversibility of the isomerization (Figure 2) [29]. In 1997, Nakashima and co-workers reported the first rotaxane where the azobenzene was located on the axle, acting as a recognition site for a β-cyclodextrin macrocycle. Photoisomerization of the azobenzene controlled the shuttling of the macrocycle [30]. Later, Stoddart and co-workers developed a rotaxane where the azobenzene was located between two phenyl rings that work as recognition sites for a CBPQT^4+^ macrocycle. Likewise, isomerization of the azobenzene modulates the shuttling of the macrocycle among the recognition sites [31]. In the same year, Anderson and co-workers reported the first [3]rotaxane with three azobenzene units in the axle and two α-cyclodextrins as the macrocycle. The rotaxane was obtained as a single stereoisomer, where the two narrow rings of the cyclodextrin point to each other. Although no photoswitching studies were conducted, ^1^H NMR results showed that for the [2]rotaxane, the macrocycle shuttles along the axle very rapidly [32].

Two decades after the first reports of azobenzene-based rotaxanes, numerous studies have been published in which azobenzene units are incorporated into the axle to control the shuttling of various macrocycles. In some systems, the azobenzene functions also as a recognition site for macrocycles such as cyclodextrins [18,33–39], or CBPQT^4+^ [40–42], while in others, it serves merely as part of the axle structure without directly participating in macrocycle recognition. Examples of the latter include systems employing CBPQT^4+^ [43], cucurbit[7]uril [44], tetralactam [45], and pillar[5]arene [46].

Moreover, Qu and co-workers synthesized [2]rotaxanes with a biphenyl and an azobenzene acting as recognition units for an α-cyclodextrin macrocycle, which has two distinct fluorescent bulky groups as stoppers. Upon both photoisomerization and thermal isomerization, the macrocycle motion along the axle is controlled, enhancing the fluorescence of the closer stopper while dimming the fluorescence of the farther stopper [47–48]. The authors later reported a [2]rotaxane containing both an azobenzene and a stilbene photoswitchable unit on the axle and an α-cyclodextrin macrocycle, where different wavelengths were required to induce isomerization of each photoswitch. Furthermore, after converting both units from *trans* to *cis*, the cyclodextrin was trapped in the middle of the axle. This reversible, light-driven shuttling of the cyclodextrin macrocycle was accompanied by alternations in fluorescence between two fluorescent stoppers [49].

**Figure 4:**
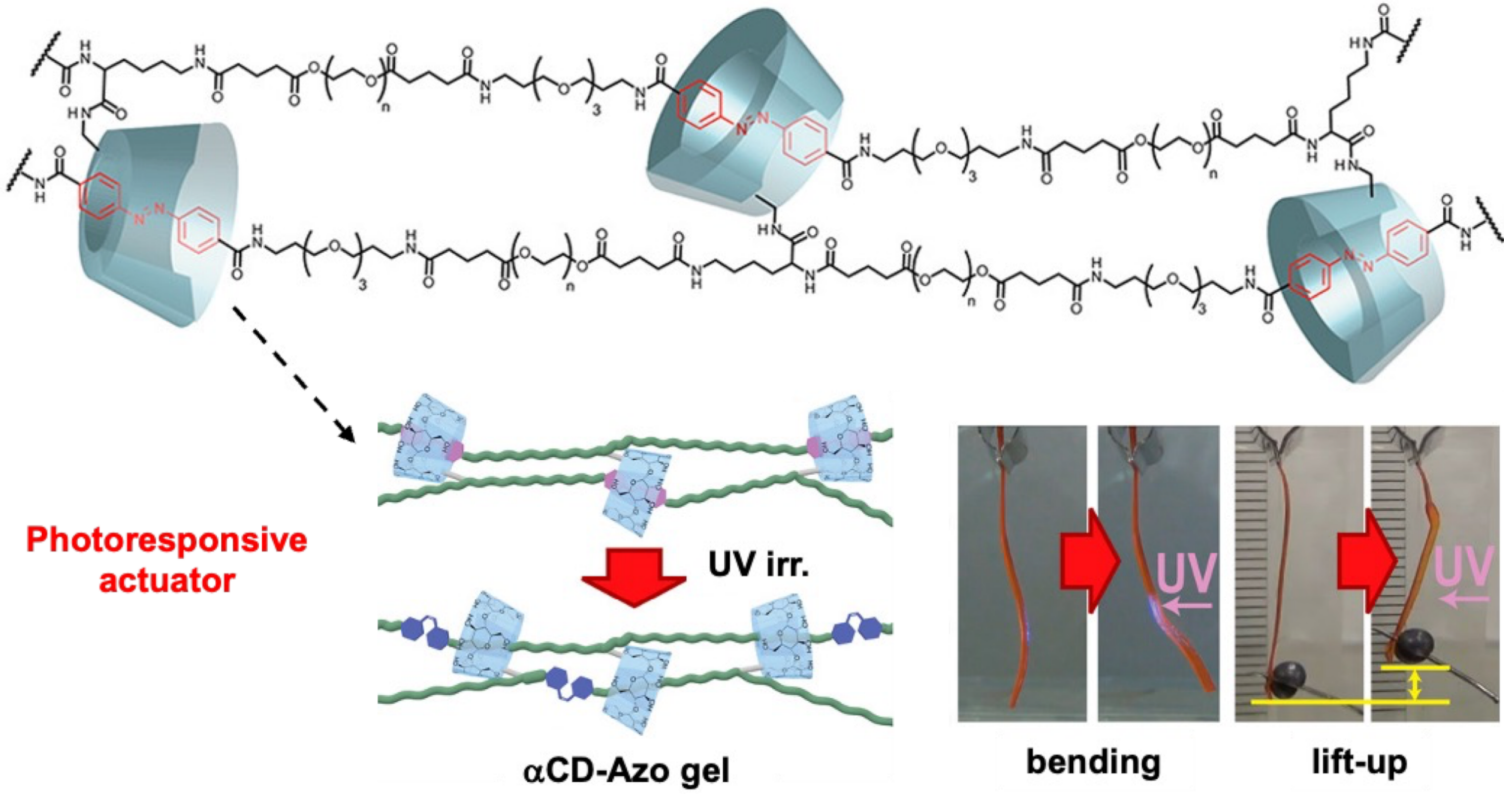
Hydrogel composed of [2]rotaxanes featuring a central azobenzene in the axle and a cyclodextrin macrocycle. (a) Light irradiation induces localized bending of the material in the same direction of the light source, and (b) demonstrates weight-lifting. Figure 4 was adapted with permission from [22], Copyright 2018 American Chemical Society. This content is not subject to CC BY 4.0.

Takashima and co-workers developed a photoresponsive polymeric actuator utilizing [2]rotaxane units as topological cross-links in a polymer network, in which the azobenzene serves as a recognition site for an α-cyclodextrin macrocycle [22]. This material demonstrated remarkable mechanical properties, including a rupture strain of 2800%, which was attributed to the stress-dispersive sliding motion of the rotaxane cross-links. Moreover, the polymeric network exhibits reversible deformation upon irradiation with UV or visible light, which triggers the isomerization of the azobenzene moiety, leading to structural changes in the rotaxane linkers. Notably, the dry state of the material, when uniaxially stretched, shows an enhanced response compared to its hydrogel form, achieving rapid mechanical actuation (Figure 4). This example illustrates the potential of topological cross-linking in advancing the field of stimuli-responsive materials, particularly in applications requiring efficient and durable artificial actuators.

**Figure 5:**
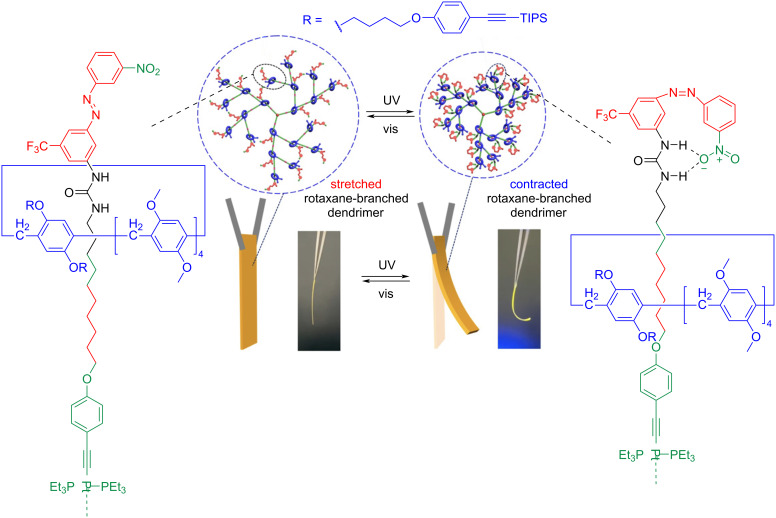
Dendrimer composed of [2]rotaxane with an azobenzene photoswitch functioning as a macroscopic actuator. Figure 5 was adapted with permission from [50], Copyright 2023 American Chemical Society. This content is not subject to CC BY 4.0.

Yang and co-workers reported a photoresponsive dendrimer that incorporates about 21 individual azobenzene-based rotaxanes serving as branches distributed within the dendrimer skeleton [50]. The [2]rotaxane consists of a pillar[5]arene macrocycle and an axle featuring a urea and alkyl chain as recognition sites. The azobenzene functioned as one of the stoppers. The molecular motion of the macrocycle during azobenzene photoswitching was utilized to demonstrate controllable and reversible shape transformation behaviors and to perform weightlifting and cargo transporting tasks (Figure 5). Therefore, these rotaxanes highlight their potential in applications as actuators in soft robotic devices.

**Figure 6:**
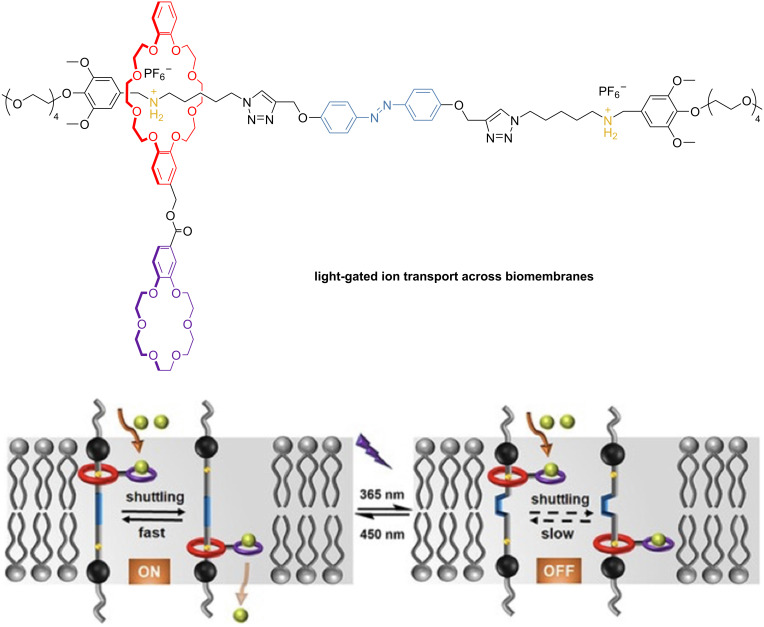
(a) Structure of the [2]rotaxane and (b) mechanism for K^+^ cations transport across lipid bilayers. Figure 6 was adapted from [51], C. Wang et al., “A Light-Operated Molecular Cable Car for Gated Ion Transport”, *Angew. Chem.*, with permission from John Wiley and Sons. Copyright © 2021 Wiley-VCH GmbH. This content is not subject to CC BY 4.0.

Wang and collaborators developed a photoswitchable [2]rotaxane featuring a central azobenzene in the axle and two benzylalkylammonium (BAA) recognition sites. As a threaded macrocycle, they used dibenzo-24-crown-8 (DB24C8), which has a smaller benzo[18]crown-6 ring appended to it, capable of capturing K^+^ ions (Figure 6) [51]. The authors demonstrated that this rotaxane can be incorporated into model lipid bilayers and mediate K^+^ ion transport across membranes through the shuttling motion of the macrocycle. In its *trans* configuration, the azobenzene allows unhindered macrocycle movement, enabling effective K^+^ ion transport. Upon photoisomerization, the *cis* isomer restricts the macrocycle’s motion and slows ion transport (Figure 6). Importantly, this switching is reversible, permitting precise light-triggered control of ion transport. Such photoswitchable and biomembrane-compatible rotaxanes are promising for designing artificial ion transporters to address channelopathies.

Song and co-workers developed a system in which the azobenzene in the axle is encapsulated within a chiral β-cyclodextrin, affording a self-locked [1]rotaxane [15]. The intramolecular host–guest interaction induces a chiral signal, while the azobenzene chromophore imparts photoswitching properties to the molecule. Furthermore, solvent-mediated hydrogen-bonding interactions tailored within the inner cyclodextrin cavity, combined with photocontrolled isomerization of the azo moiety, enable dual orthogonal manipulation of the rotaxane’s conformational regulation and chiroptical switching properties. Thus, this system functions as a phototriggered chiral on–off switch in polar aprotic solvents. These types of rotaxanes hold promise for developing simple information storage materials and anti-counterfeiting materials.

Credi and co-workers recently introduced photoswitchable axles containing azobenzene units to control the threading and dethreading of macrocycles such as crown ethers and cyclodextrins. Although these systems are pseudorotaxanes, they are noteworthy for their potential in the development of molecular pumps and ratchets [52–53].

#### Cycloheptatriene (CHT)

Cycloheptatriene is a 7-membered non-aromatic ring that can be interconverted into the corresponding tropylium ion via a photoheterolysis reaction, whereas back isomerization is achieved thermally (Figure 2). In 2004, Abraham and co-workers reported the first rotaxane containing a cycloheptatriene photoswitchable unit in a folded molecular axle and a CBQT^4+^ macrocycle [54]. The authors demonstrated that upon light exposure, the positively charged tropylium ion was generated, which repelled the CBPQT^4+^ macrocycle and modulated the co-conformation of the rotaxane. However, the folded geometry of the axle restricts long-range movement of the macrocycle. Therefore, the authors later reported a photoswitchable rotaxane with an unfolded molecular thread, achieved by modifying the stopper groups and adjusting the linker chain length. These modifications enabled large-amplitude shuttling motions and created sufficient spatial separation, ensuring the macrocycle resides exclusively at one recognition site at a time [55].

#### Dithienylethene

Dithienylethene photoswitches undergo an electrocyclic reaction, forming a closed-ring isomer upon irradiation with light. Notably, they are thermally irreversible, so the back isomerization occurs only upon irradiation with light at longer wavelengths. Although photocyclization leads to subtle structural changes (Figure 2), the two isomers present very different photochemical and photophysical properties [56]. Contrary to azobenzenes, where the isomerization modulates the position of the macrocycle, in dithienylethene-based rotaxanes, it is the shuttling of the macrocycle that modulates the fluorescence and photoswitching behavior of the dithienylethene. For example, Yin and co-workers reported in 2014 the first examples of rotaxanes with a dithienylethene switch on the axle [57]. They synthesized [3]- and [5]rotaxanes with a symmetric axle containing a dithienylethene in the center and alkylammonium binding sites on each side, which interact with *N*-hetero crown ether macrocycles. Although no shuttling or energy transfer process was observed, both rotaxanes presented good photoisomerization behavior. Remarkably, the use of cucurbit[6]uril macrocycles enhanced the photoisomerization.

Later, Liu and co-workers designed a symmetric [3]rotaxane where the pH-responsive shuttling motion of the DB24C8 macrocycles can control the photocyclization of the dithienylethene. For instance, when the DB24C8 is positioned at the *N*-methyltriazolium (MTA) site, the photocyclization rate is enhanced because the macrocycle locks the antiparallel conformer of the dithienylethene. In contrast, the opposite occurs when DB24C8 is situated further away from the dithienylethene, at the dibenzylammonium site [58]. Moreover, the authors showed that the fluorescence of the dithienylethene can also be controlled by attaching a pyrene unit to the DB24C8 macrocycle. When the dithienylethene is closed, electron transfer from the pyrene to the dithienylethene acceptor occurs, quenching its fluorescence. Moreover, faster quenching was observed when the macrocycles were closer to the dithienylethene unit [59].

**Figure 7:**
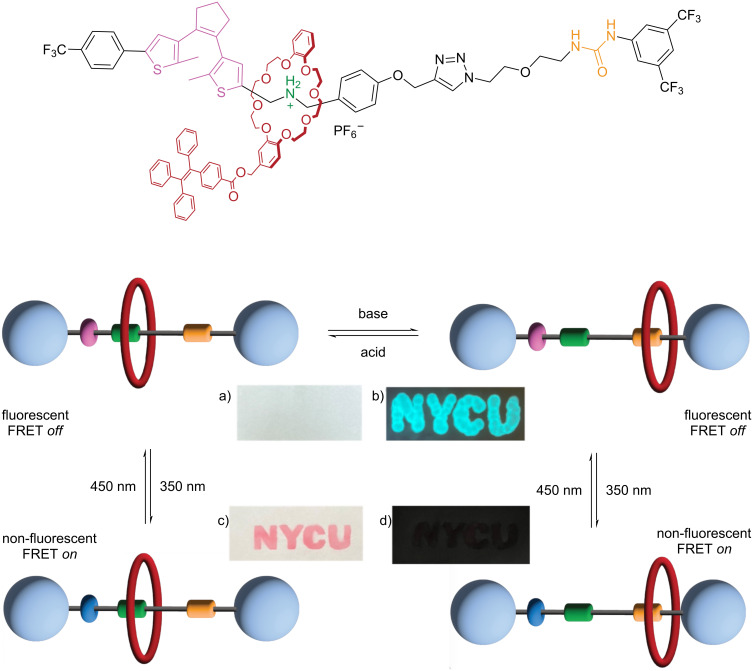
Dithienylethene-based [2]rotaxane used in writing patterning applications: (a) rotaxane with open dithienylethene while the macrocycle is located in the ammonium site under room light, (b) rotaxane with open dithienylethene while macrocycle is located in the urea site while excited at 365 nm in a dark room, (c) rotaxane with closed dithienylethene while macrocycle located in the ammonium site under room light, and (d) rotaxane with closed dithienylethene while the macrocycle is located in the urea site while excited at 365 nm in a dark room. Figure 7 was adapted with permission from [16], Copyright 2023 American Chemical Society. This content is not subject to CC BY 4.0.

Considering the discoveries on tunable fluorescence, Alene and co-workers combined the fluorescence quenching phenomena with aggregate-induced emission (AIE) in aqueous media. They synthesized a [2]rotaxane with one dithienylethene unit as a stopper, and one BAA and one urea binding site, as well as one DB24C8 macrocycle with a tetraphenylethene (TPE) fluorophore [16] (Figure 7). Addition of base results in the deprotonation of the ammonium site and shuttling of the macrocycle to the urea site. Whereas, upon addition of acid, the macrocycle returns to the protonated BAA site. Notably, the rotaxane exhibited high fluorescence intensity due to aggregation in acetonitrile with high water content, compared to pure acetonitrile. Photocyclization of the dithienylethene with UV light quenches the fluorescence of the TPE due to Förster resonance energy transfer (FRET) between the TPE (donor) and the closed dithienylethene (acceptor). Irradiation with visible light triggers the cycloreversion, regenerating the fluorescence. Notably, the application of this rotaxane in photoswitchable patterning allowed for a sequence of letters to be “erased” with UV light (Figure 7).

**Figure 8:**
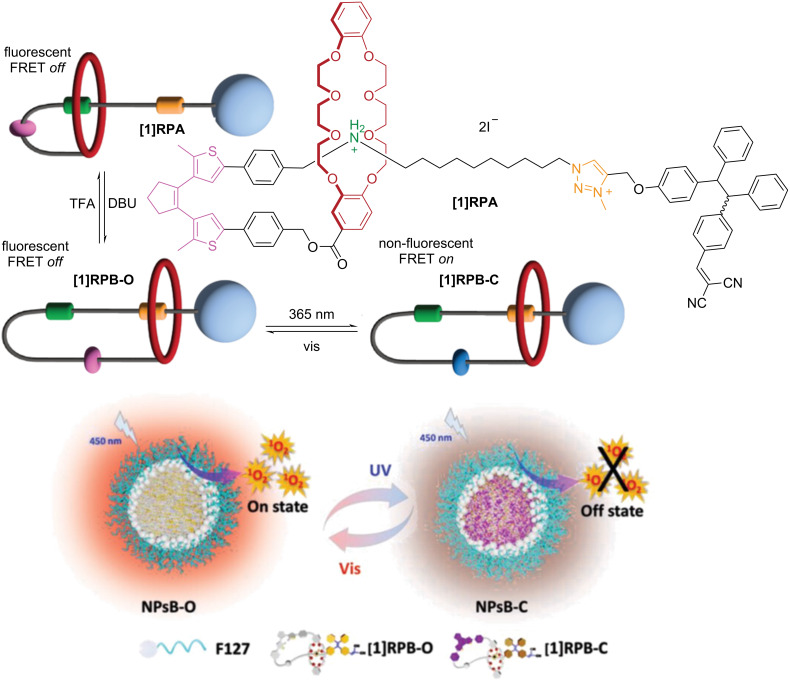
Dithienylethene-based [1]rotaxane shuttling motion triggered by pH changes (top). Dithienylethene photoswitch modulates singlet oxygen generation to kill HeLa cells (bottom). Figure 8 was adapted from [60], T. M. Khang et al., “Dual and Sequential Locked/Unlocked Photochromic Effects on FRET Controlled Singlet Oxygen Processes by Contracted/Extended Forms of Dithienylethene-Based [1]Rotaxane Nanoparticles”, *Small*, with permission from John Wiley and Sons. Copyright © 2022 Wiley-VCH GmbH. This content is not subject to CC BY 4.0.

Following similar principles, Khang and co-workers synthesized a [1]rotaxane featuring a dithienylethene photoswitch in the axle, which had a DB24C8 macrocycle on one end and a TPE unit as the stopper on the other end (Figure 8) [60]. The shuttling between the BAA (*off*) and MTA (*on*) recognition sites, induced by changes in pH, enabled the photochromism of the rotaxane to be switched on and off. Specifically, the dithienylethene cannot undergo the photocyclization reaction when the macrocycle is in the BAA site due to conformational restraint. This system was used to form water-soluble nanoparticles to investigate their biological applications. The obtained nanoparticles exhibited bright red photoluminescence and the ability to generate singlet oxygen (¹O_2_), which can be controlled through dithienylethene photoisomerization. Therefore, the authors investigated these nanoparticles for bioimaging and apoptosis of HeLa cells. When the dithienylethene is in the open form, the nanoparticles have relatively high cytotoxicity due to the in-situ generation of toxic ^1^O_2_.

#### Fumaramide

Fumaramide is a bisamide photoswitch with a thermally stable *trans*-olefin, which, upon UV light irradiation, undergoes *trans*-to-*cis* isomerization, affording the corresponding maleamide (Figure 2). These photoswitches are distinct due to their ability to form selective hydrogen bonds between the carbonyl oxygen of the fumaramide unit and the N–H groups of benzylic amide rings. Upon isomerization to the *cis* isomer, the hydrogen-bonding interactions are weakened, enabling the macrocycle to migrate to a second recognition station [61]. Despite their structural differences, fumaramide switches operate similarly to azobenzenes in rotaxanes, utilizing light-induced *trans*–*cis* isomerization to control the macrocycle shuttling [62].

Leigh and co-workers developed the first furmaramide-based rotaxanes, in which the photoswitchable unit was incorporated into the axle and allowed geometrical changes to occur, thereby modifying the nature and strength of the interaction between the macrocycle and the recognition sites [63–64]. Later, a new rotaxane was reported, which generates a strong induced circular dichroism response when the macrocycle is hydrogen-bonded to a chiral peptide station. This rotaxane works as a chiroptical switch by light-induced macrocycle movement along the axle [65]. The need to understand the photochemistry of fumaramides to aid their usage in various applications led to the design of rotaxanes containing different axles. Both experimental and theoretical studies involving these rotaxanes showed that no side reactions occur upon excitation, meaning these compounds can be operated continuously under illumination with very little efficiency loss, making them very effective photoactive units in nanodevices.

**Figure 9:**
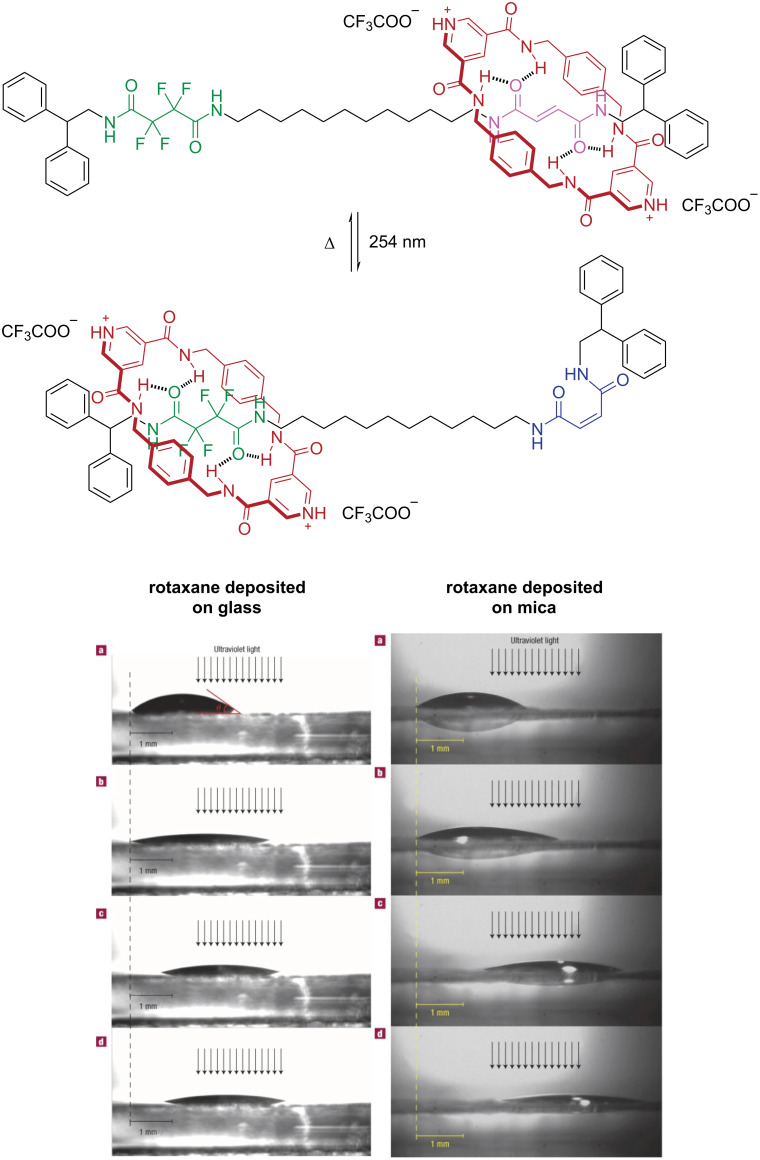
Depiction of a fumaramide-based [2]rotaxane photoswitching cycle and deposition on glass and mica surfaces for diiodomethane droplets transportation. Figure 9 was adapted from [66] (J. Berná et al., *Nat. Mater*., 2005, 4, 704, © Springer Nature 2005). This content is not subject to CC BY 4.0.

In 2005, Leigh and co-workers utilized a photoresponsive furmaramide [2]rotaxane as a macroscopic transporter for droplets of diiodomethane on the surfaces of glass and mica (Figure 9) [66]. This presents a promising method for liquid transport, with potential applications in lab-on-a-chip systems, including targeted analyte delivery and microscale chemical reactions, by allowing the controlled merging of droplets containing different reactants without the need for traditional reaction vessels.

Furthermore, Zhu and co-workers developed a rotaxane-based fluorescence switch in which photoinduced shuttling of the benzylic amide macrocycle occurs along a thread containing fumaramide and succinic amide ester units as recognition sites, with highly fluorescent perylene bisimide and pyrene serving as stoppers [67]. The macrocycle features two pyridine units that, upon protonation, effectively quench the fluorescence of both chromophores via photoinduced electron and energy transfer within the pyrene–perylene dyad. This interlocked system functions as a fluorescence switch, presenting strong potential for optical applications.

Martínez-Cuezva and co-workers synthesized photoswitchable [2]rotaxanes for catalytic applications. They incorporated a photoswitchable fumaramide and a thiodiglycolamide unit as recognition sites along the axle of the interlocked system [68]. The catalytic performance of the [2]rotaxane was assessed in a TiCl_4_-promoted Baylis–Hillman reaction between *p*-nitrobenzaldehyde and 3-butyn-2-one, where the *trans*-fumaramide showed stereoselectivity. Upon photoswitching to the *cis* isomer, the macrocycle moved to the thiodiglycolamide site, which disrupted its ability to catalyze the reaction, leading to the loss of stereoselectivity. This work represents the first evidence of a light-responsive interlocked catalyst capable of modulating reaction stereoselectivity.

#### Hydrazone

Hydrazones undergo *trans*–*cis* isomerization at the C=N bond when exposed to light, and in some instances, they can also undergo thermal isomerization (Figure 2) [69]. Although there are early reports of hydrazone derivatives in rotaxanes [70], the incorporation of functional hydrazone photoswitches into rotaxanes began more recently with Leigh. Thus, these photoswitches are underexplored in MIMs.

Specifically, Leigh and co-workers synthesized [2]rotaxanes in which a pyridyl-acyl hydrazone moiety functioned as both photo- and thermal-switchable binding site, enabling precise control over molecular motion [71]. The incorporation of pyridyl-acyl hydrazone units is demonstrated to enable high binding (>95%) and reversible macrocycle shuttling between two binding sites (98%). Moreover, structural analysis revealed distinct intercomponent hydrogen-bonding patterns that dictate the position of the macrocycle.

Later, Leigh and co-workers introduced a new strategy for dynamically controlling asymmetric catalysis using a hydrazone-based rotaxane [72]. The axle features a hydrazone photoswitch and a pseudo-*meso* 2,5-disubstituted pyrrolidine organocatalytic unit. The photoisomerization of the hydrazone modulates the position of the tetraamide macrocycle around the pyrrolidine, breaking its symmetry and switching its handedness. This dynamic control was used to adjust the enantioselectivity of an enamine-mediated conjugate addition.

**Figure 10:**
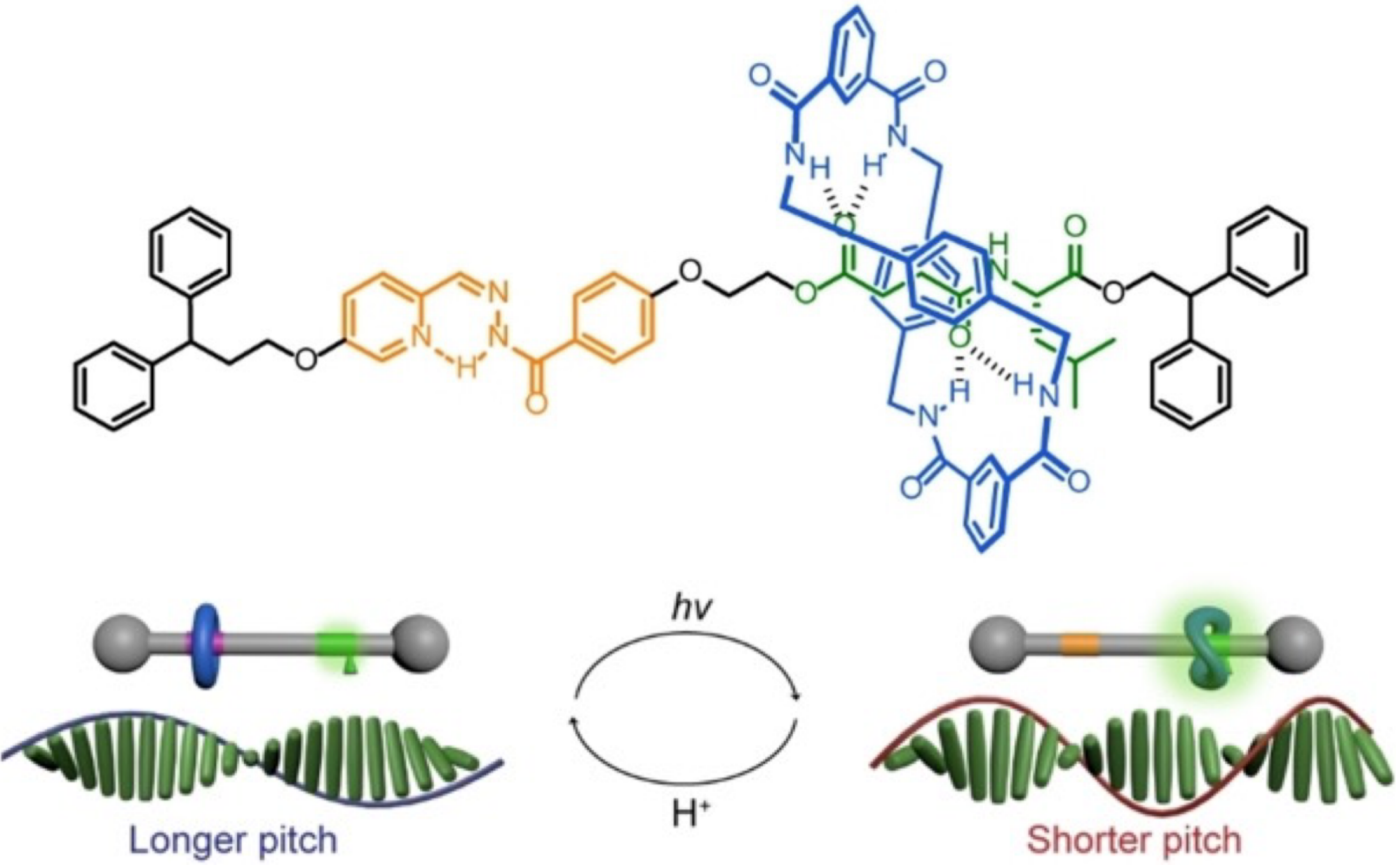
Hydrazone-based rotaxane controls helical pitch in a liquid crystal. Figure 10 was adapted from [73] (© 2024 S. Chen et al., *Angewandte Chemie* published by Wiley-VCH GmbH, distributed under the terms of the Creative Commons Attribution 4.0 International License, https://creativecommons.org/licenses/by/4.0/).

In 2024, the same group reported a [2]rotaxane with controlled shuttling motion of the macrocycle, which results in a reversible control over liquid crystal helical pitch [73]. The system features a benzylic amide macrocycle that shuttles between a pyridyl-acyl hydrazone site and a glycyl-β-leucine site via *trans–cis* photoisomerization of the hydrazone. When the macrocycle is closer to the chiral center (*cis* isomer), the rotaxane exhibits significantly enhanced chiral expression, as confirmed by a stronger circular dichroism and an increase in the helical twisting power of the liquid crystal (Figure 10). This example demonstrates that mechanical motion within a rotaxane can propagate molecular-level stereochemical information to the macroscopic organization of soft matter, providing a new strategy for dynamic chirality control in functional materials.

#### Spiropyran

When spiropyrans are irradiated with UV light, the breaking of the C–O bond “opens” the molecule, generating the corresponding merocyanine (Figure 2). The reverse photocyclization can occur upon exposure to visible light. Remarkably, the two isomers differ in their chemical and physical properties such as polarity, molecular volume, dipole moment, fluorescence emission, net charge, and color, which distinguish them from other photoswitches. Thus, incorporating these photoswitches into rotaxanes enables the modulation of the macrocycle–axle interactions, leading to materials and biological applications. The first spiropyran-based rotaxane was reported in 2007 by Zhou and co-workers [74]. The spiropyran was positioned on the axle and served a dual purpose: as a recognition site and stopper. Photoisomerization led to the reversible shuttling of the macrocycle, which produced a visible absorption output signal that can be seen with the naked eye.

Later, Lin and co-workers reported [2]rotaxanes that contain naphthalimide fluorophores on the macrocycle and a spiropyran as one of the axle stoppers [17]. The photoswitching of the spiropyran was used to modulate the photoluminescence of the rotaxane through Förster resonance energy transfer (FRET) with the naphthalimide. Specifically, red fluorescence was observed when the merocyanine formed, and green fluorescence when the spiropyran was generated. Notably, this rotaxane was employed for the detection of sulfite, where the addition of sulfite led to the deactivation of FRET. This occurs due to a Michael addition taking place at the active site (C=C−C=N^+^) in the merocyanine unit in the presence of sulfite ions, resulting in color changes from red to green. This work represents a substantial advancement in the development of rotaxane-based fluorescent probes, providing highly sensitive and selective detection of sulfite for potential biological applications.

**Figure 11:**
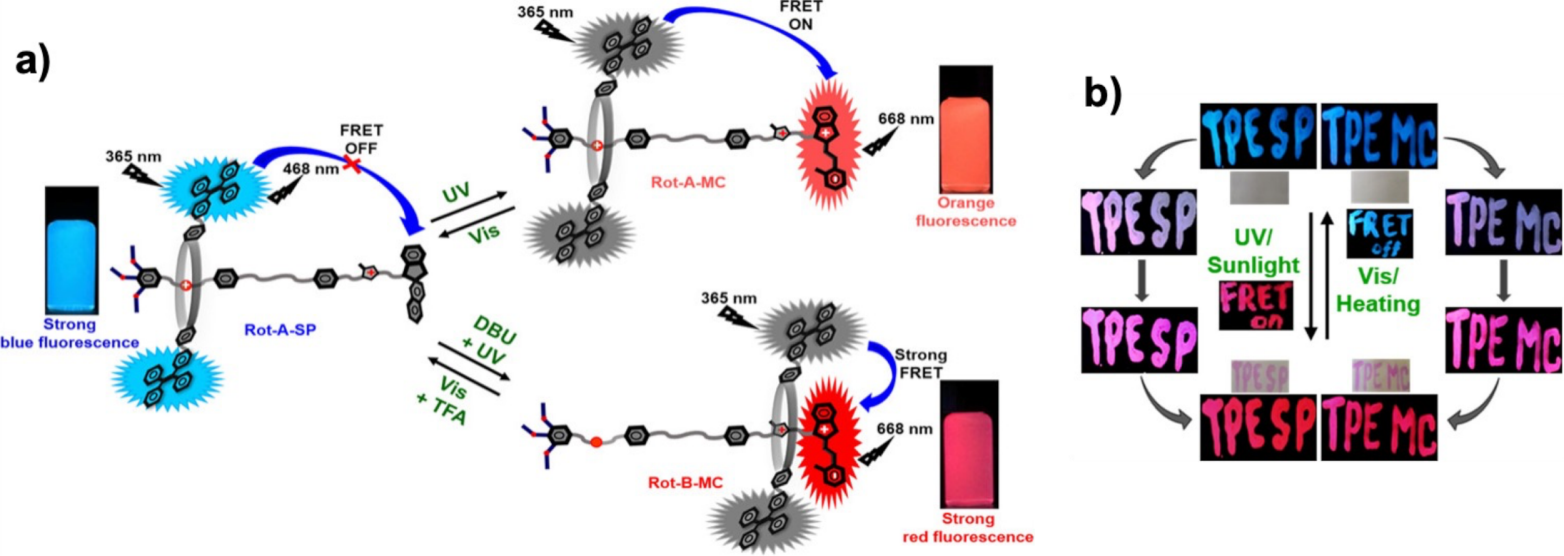
(a) Light- and pH-responsive Förster resonance energy transfer observed on a spiropyran-based [2]rotaxane and (b) its application in photopatterning. Figure 11 was adapted with permission from [20], Copyright 2020 American Chemical Society. This content is not subject to CC BY 4.0.

Furthermore, the same group reported a photoswitchable [2]rotaxane containing a spiropyran stopper and a macrocycle armed with tetraphenylethylene for photopatterning applications (Figure 11) [20]. The macrocycle has been shown to shuttle along the axle in response to pH changes, moving farther from the spiropyran upon the addition of acid. In the spiropyran form, no FRET occurs between the tetraphenylethylene fluorophore on the macrocycle (donor) and the spiropyran unit, regardless of their relative positions. However, upon photoisomerization to the merocyanine (open) form, FRET is activated – even when the macrocycle is relatively distant from the merocyanine – and becomes more pronounced as the donor approaches the acceptor. Moreover, this rotaxane exhibited tunable photoluminescence properties in response to solvent polarity, pH, temperature, and acid–base stimuli. Additionally, this spiropyran-based rotaxane showed pronounced photochromic and fluorescent behavior in both powder and solid film under alternating UV/sunlight and visible light/heating conditions, highlighting its suitability for dynamic optical systems and anti-counterfeiting applications.

#### Stilbene and stiff-stilbene

Stilbene photoswitches consist of a central olefin with a phenyl group at each end. Upon light irradiation, they undergo *trans*-to-*cis* isomerization, with the reverse isomerization triggered photochemically at a different wavelength or thermally (Figure 2). However, the *cis* isomer may undergo a 6π-electrocyclization, forming dihydrophenanthrene intermediates that further oxidize into phenanthrene derivatives, thereby irreversibly deactivating the photoswitch [75].

The first stilbene-based rotaxane was reported by Anderson and co-workers in 2001, featuring a stilbene unit incorporated into the axle, which served as a recognition site for a cyclodextrin macrocycle [76]. However, in this initial study, the authors only investigated how the fluorescence properties of the stilbene were affected by its confinement in the cyclodextrin. Soon thereafter, the same group reported related [2]rotaxanes in which stilbene *trans-*to*-cis* photoisomerization induced the translocation of the cyclodextrin along the axle [77]. Remarkably, the movement of the macrocycle was unidirectional, driven by the asymmetric size difference between the two rims of the cyclodextrin.

**Figure 12:**
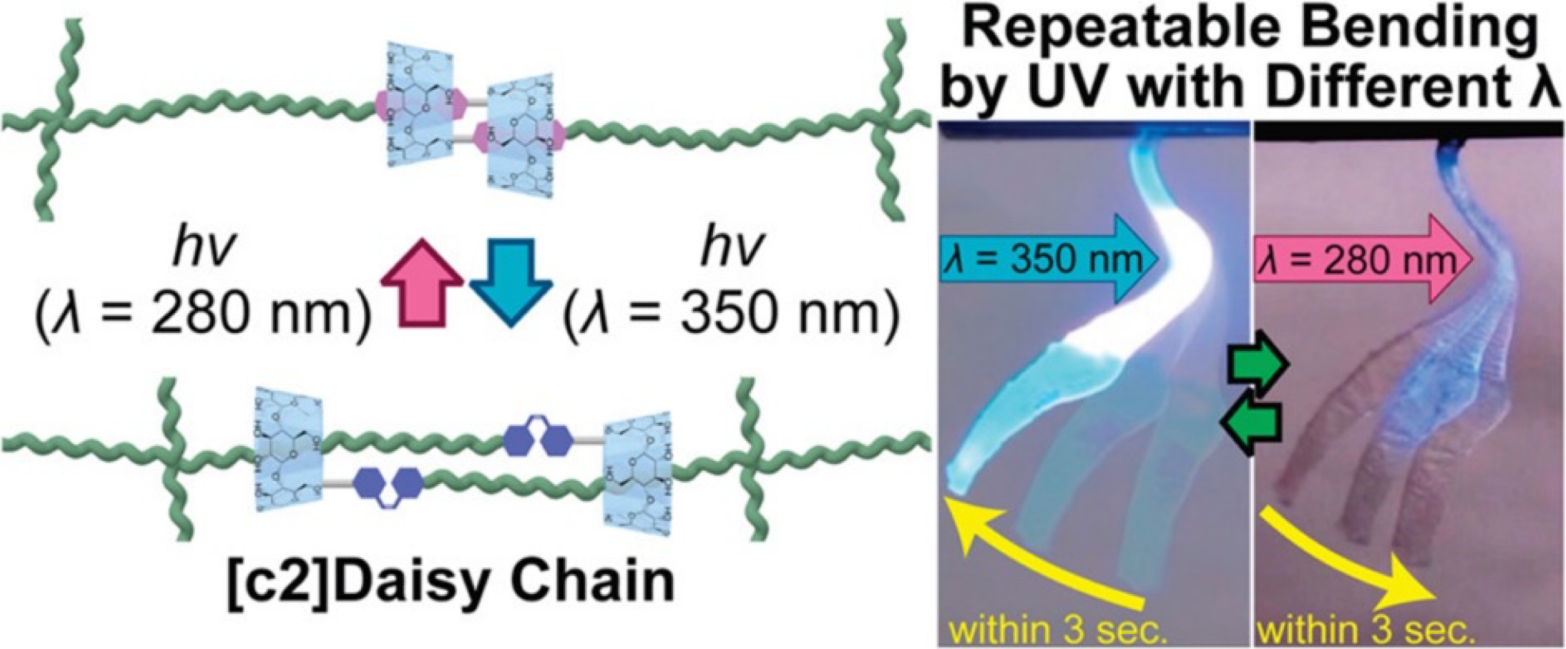
Photoresponsive bending of artificial muscle with [c2]daisy chain reported by Harada and collaborators. Figure 12 was adapted with permission from [78], Copyright 2018 American Chemical Society. This content is not subject to CC BY 4.0.

Harada and co-workers demonstrated the potential of stilbene-based rotaxanes by developing artificial muscles [78]. They created gels based on [c2]daisy chains, which are double-threaded [2]rotaxane dimers with stilbene as the recognition unit for a cyclodextrin macrocycle (Figure 12). The photoisomerization of the stilbenes caused the cyclodextrin to displace along the axle, resulting in rapid and significant contraction or expansion of the gels. Moreover, stilbene photoisomerization was used to perform bending motions and lift a weight 15 times heavier than the dry gel.

Contrary to stilbenes, stiff-stilbenes have 5-membered rings fused to the carbon–carbon double bond, are more photostable, and undergo reversible photoisomerization with higher efficiency (Figure 2) [75]. Notably, their *cis* isomer exhibits higher thermal stability than that of other commonly used photoswitches, such as azobenzene. This property, in addition to facilitating their study and isolation, is advantageous for various applications.

The first stiff-stilbene-based rotaxane was reported in 2018 [79]. This [1]rotaxane used stiff-stilbene as one of the stoppers, and its photoisomerization produced the translation of a pillar[5]arene macrocycle along a hydrophobic axle with a central urea unit. More recently, a rotaxane incorporating a stiff-stilbene unit at the center of the axle was synthesized, featuring two DB24C8 macrocycles primarily positioned at quaternary ammonium recognition sites [80]. In this system, the *trans* isomer of the stiff-stilbene facilitated the formation of aggregates, resulting in organic room-temperature phosphorescence. The phosphorescent properties can be modulated by inducing macrocycle translation through acid–base stimuli or by disrupting the aggregates via stiff-stilbene photoisomerization. The authors demonstrated that this system can be utilized in information encryption, anti-counterfeiting, and photorecording.

**Figure 13:**
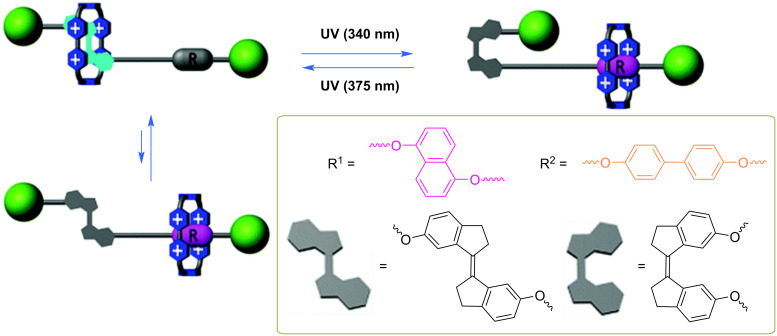
Light-responsive shuttling motion of [2]rotaxane based on a stiff-stilbene photoswitch. Figure 13 was reproduced with permission of The Royal Society of Chemistry, from [81] (“Toward bidirectional photoswitchable colored photochromic molecules with visible light stability” by T.-G. Zhan et al., *Chem. Commun.*, vol. 54, issue 67, © 2018); permission conveyed through Copyright Clearance Center, Inc. This content is not subject to CC BY 4.0.

Zhan and co-workers synthesized a photochromic bistable [2]rotaxane in which the *trans*-stiff-stilbene served as the primary recognition unit for a CBPQT^4+^ macrocycle (Figure 13) [81]. The photoisomerization to the *cis* isomer caused the macrocycle to shift to a secondary recognition unit, either a 1,5-dioxynaphthalene or a biphenyl, resulting in a significant color change, a desired property for information storage.

### Rotaxanes with different switchable units on the macrocycle

Rotaxanes incorporating photoswitchable units directly into the macrocycle remain relatively underexplored compared to their axle-based counterparts. This likely stems from the long-standing focus on the shuttling motion of the macrocycle as the hallmark of rotaxane function, which naturally led to early designs positioning photoswitches on the axle to control macrocycle movement. However, recent advances have revealed that embedding photoswitchable units within the macrocycle unlocks new and exciting functional possibilities as discussed below.

#### Acridane

Abraham and co-workers attempted to synthesize a [2]rotaxane incorporating an acridane photoswitch directly into a crown ether macrocycle [82]. However, the synthesis proceeded with very low yield, and the small amount of rotaxane obtained was immediately converted to the acridinium form during purification. Attempts to regenerate the acridane were unsuccessful. Nevertheless, the authors successfully prepared the corresponding pseudorotaxane (lacking stopper groups), which underwent dethreading upon light activation, demonstrating the potential of acridane-based macrocycles for light-controlled mechanical motion in rotaxanes.

#### Anthracene

Hirose and co-workers developed [2]rotaxanes composed of a large crown ether macrocycle containing two anthracene moieties and an axle consisting of dibenzylammonium hexafluorophosphate [83]. Light-induced anthracene dimerization reduces the macrocycle’s cavity, strengthening its interaction with the axle. As a result, the initially rapid dethreading is suppressed, and the macrocycle becomes effectively immobilized. The process is reversible upon heating. Later, Tron and co-workers synthesized a [2]rotaxane containing a barbiturate moiety in the axle, which serves as a template for a reversible clipping approach [84]. The macrocycle precursor is an acyclic Hamilton-like receptor with terminal anthracene moieties on both ends. Therefore, upon irradiation with light, the anthracene undergoes photodimerization, forming a macrocycle and capturing the axle to afford the [2]rotaxane. Notably, the photoclipping approach is thermally reversible.

#### Azobenzene

**Figure 14:**
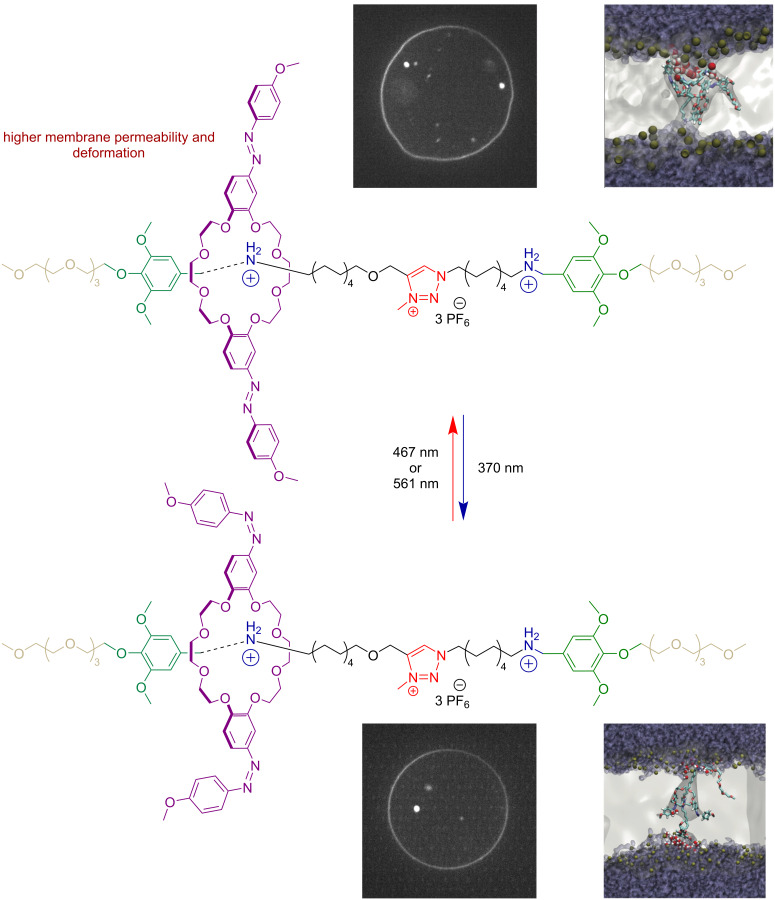
Azobenzene-based rotaxane modulating lipid bilayers upon photoisomerization. Figure 14 was adapted from [23] (© 2024 U. N. K. Conthagamage et al., published by Springer Nature, distributed under the terms of the Creative Commons Attribution-NonCommercial-NoDerivatives 4.0 International License, https://creativecommons.org/licenses/by-nc-nd/4.0/). This content is not subject to CC BY 4.0.

Recently, the García-López group reported the first rotaxane featuring two azobenzene photoswitches integrated into a DB24C8 macrocycle (Figure 14) [23]. The azobenzene units retained their excellent photoswitching performance and photostability. In the *tran*s isomer, the macrocycle exhibited shuttling motion along a 3.2 nm axle between two BAA recognition sites in acetonitrile solution. The behavior of this rotaxane was investigated in lipid bilayers of large and giant vesicles. Remarkably, the photoisomerization of the azobenzene units enabled modulation of the rotaxane's interaction with the lipid membrane, leading to reversible changes in giant lipid vesicles. In the *trans* isomer, the membrane tension decreased, resulting in deformation and pronounced membrane undulations. Isomerization to the *cis* form increased membrane tension, causing vesicle contraction. Molecular dynamics simulations revealed that while azobenzene photoisomerization can directly induce some degree of lipid packing reorganization, the dominant effect arises from changes in the position of the rotaxane within the membrane. Specifically, in the *trans* isomer, the macrocycle resides closer to the membrane–water interface, promoting local water accumulation and membrane permeabilization. In contrast, in the *cis* isomer, the macrocycle penetrates deeper into the hydrophobic core of the bilayer, reducing perturbation and water accumulation, thereby increasing membrane tension.

Light-driven membrane modulation holds great promise for drug delivery, therapeutics, and various biotechnological applications [85–88]. As a proof of principle, this rotaxane was used to trigger the light-induced release of hydrophilic cargo from large vesicles, highlighting its potential as a platform for controlled drug delivery.

Recently, Yang and co-workers reported a [2]rotaxane featuring a macrocycle constructed from two azobenzene photoswitches threaded onto an asymmetric axle [89]. Photoisomerization of the azobenzenes alters the geometry and size of the macrocycle, thereby modulating its affinity toward two distinct recognition sites on the axle – an ammonium and a pyridinium group. In the all-*trans* state, the macrocycle preferentially associates with the pyridinium site, whereas in the *cis* state it shuttles to the ammonium residue. Back isomerization, triggered either thermally or with light, returns the macrocycle to the pyridinium site. This light-driven shuttling was shown to be fatigue-resistant over multiple cycles, highlighting the robustness of the system. Notably, the authors also reported a general quantification of kinetic asymmetry in a multicycle chemical reaction network, establishing a framework applicable to other molecular systems.

#### Dithienylethene

**Figure 15:**
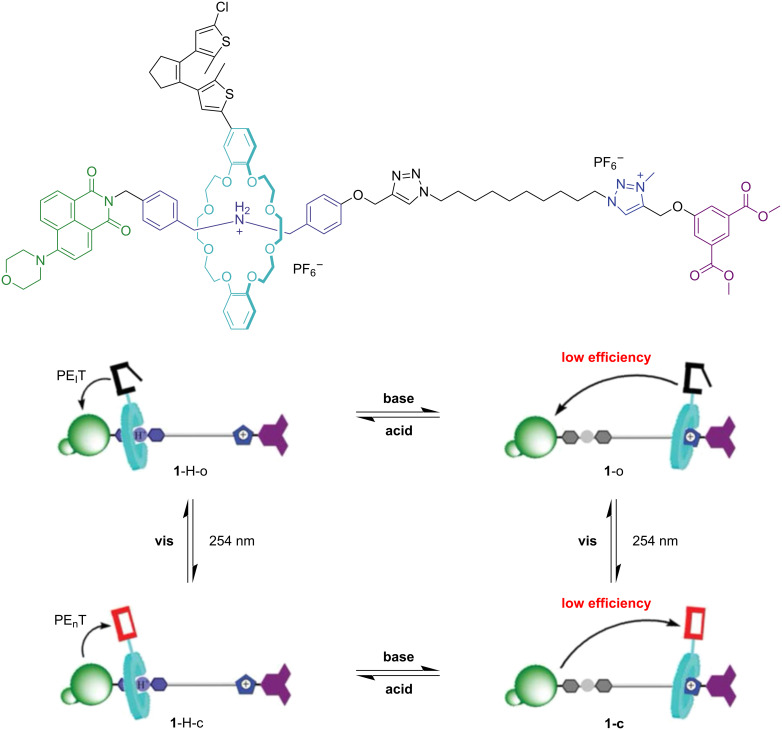
Depiction of fluorescence quenching processes upon external stimuli of a dithienylethene-based [2]rotaxane. Figure 15 was adapted with permission of The Royal Society of Chemistry, from [90] (“Altering intercomponent interactions in a photochromic multi-state [2]rotaxane” by H. Zhang et al., *Org. Biomol. Chem.*, vol. 9, issue 11, © 2011); permission conveyed through Copyright Clearance Center, Inc. This content is not subject to CC BY 4.0.

Incorporation of dithienylethene switches on the macrocycle of rotaxanes has been done primarily to modulate the fluorescence properties of the system. Tian and co-workers reported the first example of this type of rotaxane, followed by Li and co-workers [90–92]. The systems consist of [2]- and [3]rotaxanes where the dithienylethene units are directly attached to either *N*-hetero crown ether or DB24C8 macrocycles. BAA and MTA units served as recognition sites for the macrocycles. Notably, Tian’s rotaxane showed fluorescence quenching when the dithienylethene is in the closed form, caused by photoinduced electron transfer (PET) from the fluorescent pyrene stopper to the acceptor dithienylethene (Figure 15). Moreover, the efficiency of the PET is modulated by the macrocycle shuttling motion between the two recognition sites due to differences in proximity to the fluorescent stopper. The reported findings involving dithienylethene-based rotaxanes prove their promising future in materials applications, mainly due to their high photofatigue resistance.

#### Spiropyran

**Figure 16:**
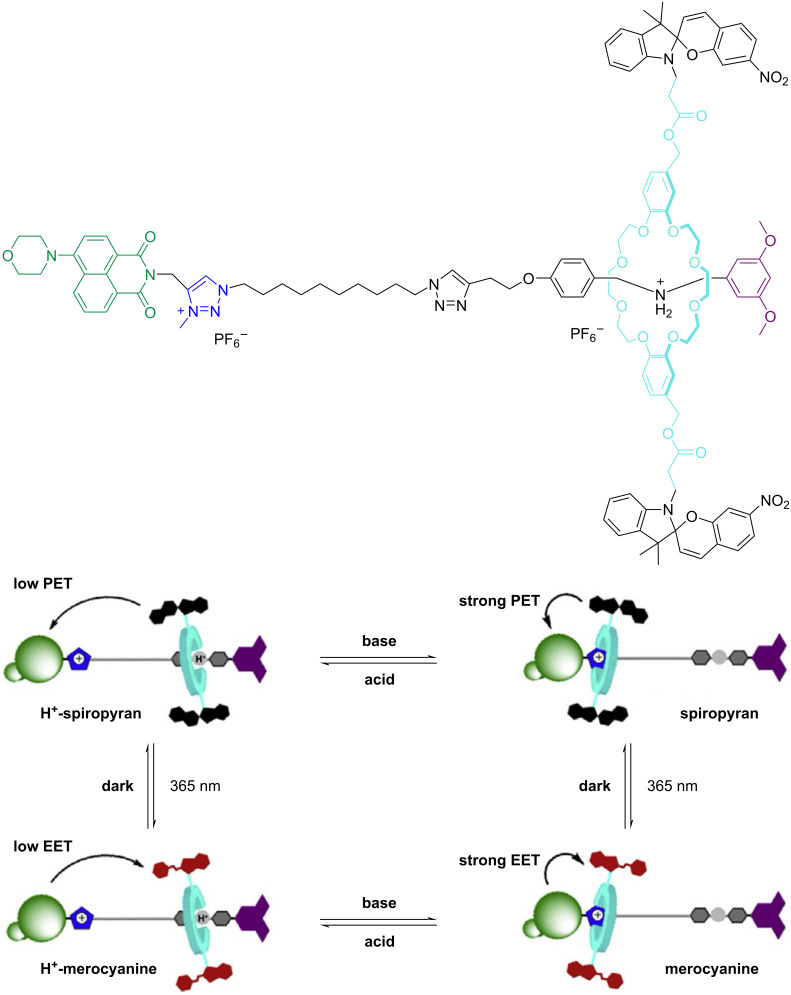
Diagrammatic illustration of rotaxane 1-H-SP depicting interconversions between the four isomeric states in response to both pH and light stimuli. Figure 16 was adapted from [93], *Tetrahedron*, vol. 69, by W. Zhou, H. Zhang; H. Li; Y. Zhang; Q.-C. Wang; D.-H. Qu, “A bis-spiropyran-containing multi-state [2]rotaxane with fluorescence output“, pages 5319–5325, Copyright (2013), with permission from Elsevier. This content is not subject to CC BY 4.0.

While most spiropyran-based rotaxanes have the photoswitchable unit located on the axle, an exception has been reported by Zhou and co-workers, where the authors designed and synthesized a [2]rotaxane containing two spiropyran units located on the macrocycle. A 4-morpholin-naphthalimide fluorophore was used as one stopper on the axle [93]. The rotaxane exhibits reversible macrocycle shuttling upon pH changes while photoisomerization of the spiropyran led to the merocyanine. Thus, four different configurational states of the interlocked system were generated, which produce distinct fluorescence outputs (Figure 16). Therefore, this work presents a multistate [2]rotaxane with light and pH-responsive fluorescence, highlighting its promising ability to design responsive optical materials and molecular logic gates.

#### Stiff-stilbene

**Figure 17:**
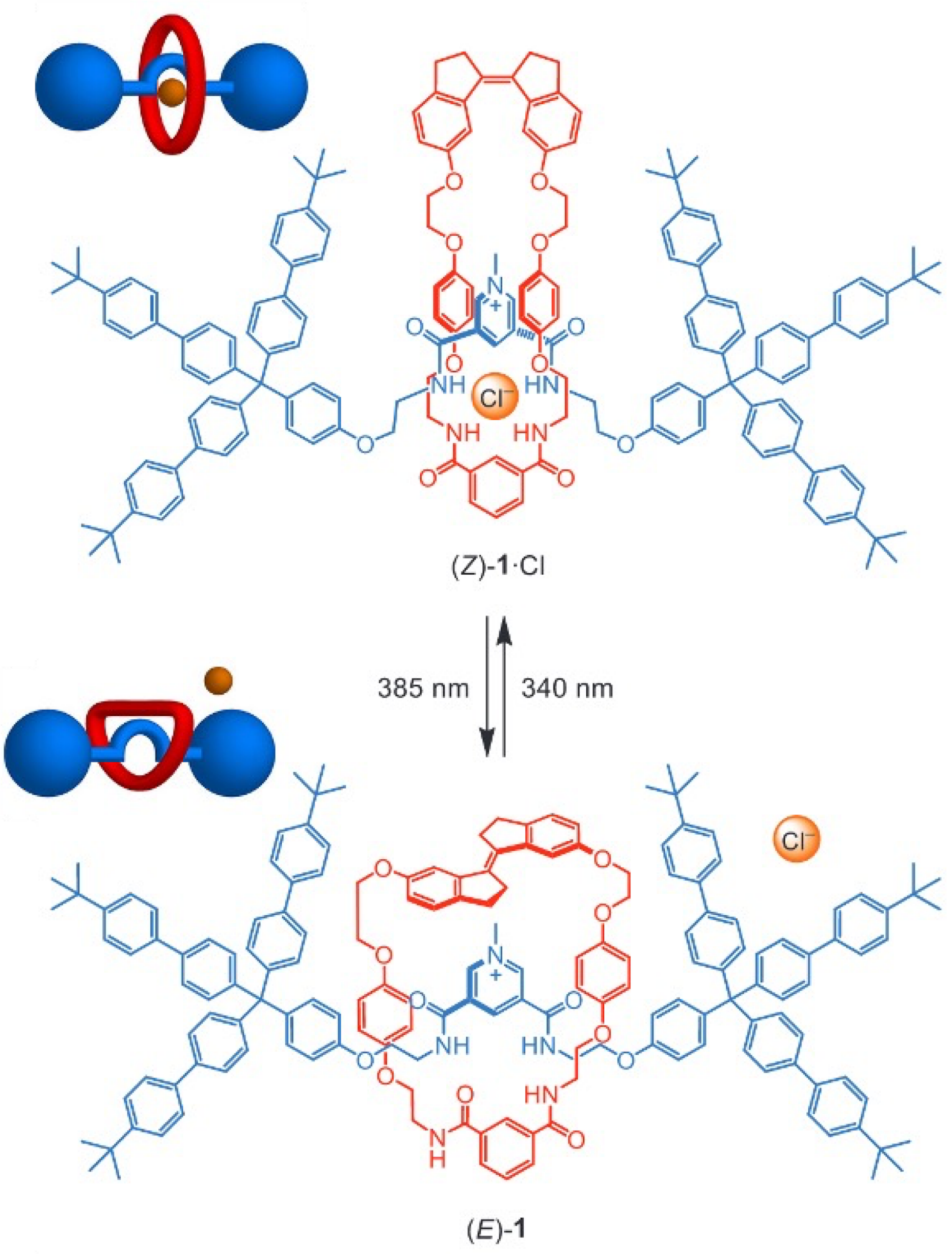
Representation of [2]rotaxane chloride binding modulated by photoisomerization of a stiff-stilbene. Figure 17 was reproduced from [95] (© 2025 J. de Jong et al., *Chemistry – A European Journal* published by Wiley-VCH GmbH, distributed under the terms of the Creative Commons Attribution 4.0 International License, https://creativecommons.org/licenses/by/4.0/).

Wezenberg and co-workers designed a photoswitchable macrocycle that incorporates a stiff-stilbene unit on one side and an isophthalamide moiety on the other [94]. The photoisomerization of the stiff-stilbene allows the macrocycle to adopt a narrow-elongated conformation in the *cis* state and a wider geometry in the *trans* state. This light-induced conformational change was utilized to control the anion-templated formation of a pseudorotaxane. Specifically, the axle contains a pyridinium bisamide that forms a complex with the isophthalamide unit of the macrocycle and a chloride anion when the stiff-stilbene is in the *cis* configuration (Figure 17). Upon switching to the *trans* isomer, the expanded geometry of the macrocycle disrupts the complex, resulting in the dethreading of the macrocycle. By introducing stopper groups to prevent dethreading, the authors demonstrated that the photoisomerization of stiff-stilbene can reversibly modulate the chloride anion binding affinity of the system [95]. Due to its selectivity and light-controlled binding behavior, this system holds potential for applications in anion transport, extraction, and separation technologies.

## Conclusion and Perspectives

The development of photoswitchable rotaxanes has mainly focused on controlling the position and movement of the macrocycle along the axle. Consequently, the vast majority of systems reported to date incorporate photoswitches into the axle to regulate shuttling dynamics. However, emerging strategies that integrate photoswitches directly into the macrocycle are creating alternative avenues for modulating not only macrocycle–axle interactions but also the interactions of rotaxanes with their surrounding environment.

Controlling macrocycle shuttling via light activation has proven valuable for enabling secondary functions. For example, when integrated into polymers or gels, the mechanical motion of rotaxanes can be harnessed to control the macroscopic properties of materials or even generate mechanical work, such as lifting objects. In other systems, photoswitchable rotaxanes have been used to modulate optoelectronic properties, creating opportunities for applications in information storage, sensing, optical devices, and anti-counterfeiting technologies.

Recently, the incorporation of photoswitchable rotaxanes into lipid membranes has revealed exciting opportunities for future biooriented applications. These applications include light-controlled systems for drug release, ion transport, rotaxane-based therapeutics, and molecular tools capable of modulating biological processes in living cells. However, most photoswitchable rotaxanes developed to date have been designed for use in organic solvents or non-biological environments. To fully harness their potential in biomedical and biotechnological settings, it is essential to establish new design principles that ensure biocompatibility, stability, and functionality under physiological conditions.

Looking forward, incorporating newer and more efficient photoswitches, along with advancements in rotaxane architecture, will expand the functional landscape of these systems. Opportunities arise not only for more sophisticated molecular machines for fundamental studies but also for practical applications in smart materials, responsive systems, and biomedical technologies. The synergy between molecular design, light-driven control, and functional integration positions photoswitchable rotaxanes as a highly versatile and dynamic platform for future innovations across multiple disciplines.

## Data Availability

Data sharing is not applicable as no new data was generated or analyzed in this study.
